# Factors influencing bedtime procrastination in junior college nursing students: a cross-sectional study

**DOI:** 10.1186/s12912-022-00881-7

**Published:** 2022-04-27

**Authors:** Dong Chen, Yuhuan Zhang, Jie Lin, Dong Pang, Dongyang Cheng, Daiwei Si

**Affiliations:** 1 Department of Nursing, Heilongjiang Nursing College, Harbin, 150086 Heilongjiang China; 2 Student affairs office, The Second Affiliated Hospital of Harbin Medical University, Harbin, 150086 Heilongjiang China; 3 Department of Neurology, The Second Affiliated Hospital of Harbin Medical University, Harbin, 150086 Heilongjiang China; 4 Department of orthopaedic, The Second Affiliated Hospital of Harbin Medical University, Harbin, 150086 Heilongjiang China

**Keywords:** Junior college nursing students, Bedtime procrastination, Personality, Self-regulation fatigue; future time perspective, Problematic Mobile phone use

## Abstract

**Background:**

Sleep quality is related to physical and mental health. Though bedtime procrastination has been identified as a potentially key source of poor sleep quality, related research is scarce. The goal of our study was to determine bedtime procrastination among nursing students and identify its influencing factors.

**Methods:**

This cross-sectional study comprised 1827 junior college nursing students. The data were collected from November to December 2021 using a mobile app-based survey. We evaluated demographic factors, Big Five personality traits, self-regulatory fatigue, future time perspective, and problematic mobile phone use. Multiple linear regression analysis was used to identify independent characteristics that influence bedtime procrastination among junior college nursing students.

**Results:**

The mean bedtime procrastination score in junior college nursing students was 25.11 ± 6.88. Family monthly income of 3000–6000 RMB (β = 0.740; *p* = 0.015), as well as that of > 6000 RMB (β = 1.708; *p* = 0.001), and an extroverted personality (β = 0.225; *p* = 0.001), self-regulatory fatigue (β = 0.135; *p* < 0.001), and problematic mobile phone use (β = 0.078; *p* < 0.001) had significant positive effects on bedtime procrastination. Conscientious personality (β = − 0.284; *p* = 0.003), neurotic personality (β = − 0.203; *p* = 0.031), and future time perspective (β = − 0.141; *p* < 0.001) had significant negative effects on bedtime procrastination.

**Conclusion:**

The nursing students who participated in this study had moderate levels of bedtime procrastination. Bedtime procrastination was predicted by higher monthly household income; personality traits of extroversion, conscientiousness, and neuroticism; self-regulatory fatigue; future time perspective; and problematic mobile phone use.

**Practical implications:**

We recommend that effective measures are needed to help alleviate bedtime procrastination and improve the health and well-being of nursing students.

**Supplementary Information:**

The online version contains supplementary material available at 10.1186/s12912-022-00881-7.

## Introduction

A good night’s sleep is critical for physical and mental wellness. However, research has revealed that nursing students tend to have poor sleep quality [1]. A previous systematic review [2] of 17 studies involving 13,247 nursing students found that the prevalence of sleep disorders among nursing students during the COVID-19 pandemic ranged from 4.4 to 52.71%, with a prevalence of sleep disorders among nursing students in India of 4.4% [3], in Turkey of 28.26% [4], in the United States of 32% [5], and in Nepal of 52.71% [6]. Poor sleep quality directly affects nursing students’ physical health [7] in a manner similar to unhealthy habits like skipping breakfast or eating late-night snacks [8]. Poor sleep quality also endangers mental health, causing anxiety [9] and depression [10], as well as sleepiness during the day [11], which affects long-term academic performance [12].

Previous research has found that the main causes of poor sleep quality in nursing students are insomnia [13] and smartphone addiction [14]. Recent research [15], however, suggests that bedtime procrastination may be the true cause of decreased sleep duration and, as a result, also of poor sleep quality. Bedtime procrastination is defined as an individual’s inability to go to bed at the scheduled time in the absence of an external impediment [16]. Individuals with bedtime procrastination, such as those with general procrastination, will delay going to bed in the absence of a valid reason for the delay and despite anticipation of a negative outcome [17]. Bedtime procrastination can cause poor sleep quality as well as negative emotions such as anxiety [18] and depression [19].

Previous research has found that bedtime procrastination is associated with individual self-control failure [20]. Personality [21] and problematic mobile phone use [22] may be influencing factors in bedtime procrastination. However, to our knowledge, there is very limited research on bedtime procrastination among nursing students. The goal of this study was to examine the current state of bedtime procrastination among nursing students and its influencing factors, as well as to provide a scientific foundation for improving sleep quality among nursing students.

## Methods

### Settings and participants

We conducted a cross-sectional study to assess bedtime procrastination among nursing students and influencing factors. In Chinese higher education, high school graduates can pursue a junior college degree in a program lasting 3 years. From November to December 2021, we selected 52 junior college nursing classes from a medical college in Heilongjiang Province, Northeast China using a convenience sampling method. The inclusion criteria were: (a) being a current junior college nursing student; and (b) signing the electronic informed consent to participate in the study. The exclusion criteria were individuals who were in suspension of schooling. In this study, we used multiple linear regression analysis, and the estimated number of influencing factors was 14. Considering a 15% nonresponse rate, the sample size was calculated to be 81–162 based on the fact that the sample size in multiple linear regression analysis should be 5 to 10 times the number of factors studied.

Owing to the COVID-19 pandemic, this cross-sectional study was conducted in the form of an Internet-based questionnaire via a mobile phone app (https://www.wjx.cn/vj/PRuTQS0.aspx), in which the purpose and importance of the study were explained. Respondents were asked to read the instructions and were given the opportunity to decide whether to accept or decline the survey. Anonymity and confidentiality were guaranteed.

Before the investigation, the researchers contacted the teachers or counsellors, and the questionnaire was also distributed (Additional file 1). Each participant was only allowed to submit one survey to avoid double submission.

### Instruments

- The general information questionnaire in this study addressed sociodemographic characteristics, including sex, age, grade, family location, number of sibling, and monthly household income.
- Bedtime Procrastination Scale (BPS). Bedtime procrastination was measured using the BPS: a brief, self-rated questionnaire that is extensively applied to assess bedtime procrastination among students. Zhang translated and amended the scale developed by Kroese [23] and created a Chinese version [24]. Response options range from “never” (1 point) to “always” (5 points). There are four entries scored in reverse. The higher the score, the worse the bedtime procrastination. Cronbach’s α coefficient of the total scale is 0.88. In our study, the Cronbach’s α was 0.834.
- Ten-Item Personality Inventory-Chinese (TIPI-C). Personality was measured using the TIPI, a well-established measure to assess personality developed by Gosling [25]. The Chinese version of the questionnaire (TIPI-C) was translated and amended by Li [26]. The Chinese version of the questionnaire has 10 items and five subscales: extroversion, agreeableness, conscientiousness, neuroticism, and openness. Response options range from “absolutely disagree” (1 point) to “absolutely agree” (7 points). There are five entries scored in reverse. The higher the score, the more obvious the corresponding personality. The Cronbach’s α coefficient of each subscale is more than 0.6, indicating good homogeneity and internal consistency.
- Self-Regulatory Fatigue Scale (SRF-S). Wang translated and amended the SRF-S scale developed by Nes [27] and created a Chinese version for use in students, which has been suggested to be a valid measurement with excellent reliability and validity [28]. The amended scale has three dimensions—cognition, emotion, and behavior—that can reflect different aspects of a depleted condition. Response options range from “absolutely disagree” (1 point) to “absolutely agree” (5 points). There are five entries scored in reverse. Higher scores reflect chronic self-regulation resource depletion or a scarcity of self-regulatory resources. With 16 entries, the Cronbach’s α coefficient for the SRF-S is 0.84. In our study, the Cronbach’s α was 0.852.
- Future time Perspective scale (FPS). Future time perspective was measured using the FPS, which was developed by Zimbardo [29]. The Chinese version of the FPS was revised by Song [30]. This questionnaire includes 20 items in five subscales: behavioral commitment, far-reaching goal orientation, future efficacy, future purpose consciousness, and future image. Response options range from “completely noncompliant” (1 point) to “fully compliant” (4 points). There are five entries scored in reverse. The higher the score, the better the future time perspective. The Cronbach’s α coefficient for the FPS is 0.793. In our study, the Cronbach’s α was 0.840.
- Self-rating Questionnaire for Adolescent Problematic Mobile Phone Use (SQAPMPU). To evaluate the level of problematic mobile phone use among junior college nursing students, we used the SQAPMPU in this study. Tao translated and amended the scale developed by Billieux [31] and created a Chinese version. The Chinese version of the SQAPMPU has been demonstrated to have excellent reliability and validity [32]. This questionnaire has 13 items and three subscales: thirst, withdrawal, and reaction of body and mind. Response options range from “never” (1 point) to “always” (5 points). The higher the score, the worse the problematic mobile phone use. The Cronbach’s α coefficient for the SQAPMPU is 0.87. In our study, the Cronbach’s α was 0.958.

### Data collection and analysis

After the questionnaires were collected, researchers conducted repeated inspections and screenings to eliminate questionnaires that took less than 2 min to complete, had irregular answers, and were logically contradictory. This study initially included 2067 junior college nursing students. After data cleaning and the deletion of data that did not meet the requirements, 1827 junior college nursing students were eventually included in the study.

All data were analyzed using IBM SPSS 25.0 (IBM Corporation, Armonk, USA). The mean (standard deviation, SD) was used to describe continuous data. Categorical variables are presented as frequency (n) and percentage. Normality of the distribution of variables was verified using the Shapiro–Wilk test. An independent samples *t*-test and one-way analysis of variance were used to compare bedtime procrastination in junior college nursing students with different demographic characteristics. The correlation between continuous variables was tested using Pearson correlation analysis. Multiple linear regression (entry method) was used for multivariate analysis due to dummy variables. In the current study, the *p* value was two-tailed, and we inferred statistical significance if α was less than 0.05.

## Results

### Demographic characteristics and bedtime procrastination of junior college nursing students

Of the 2067 samples invited, 1827 submitted valid surveys, so the effective rate was 88.39%. The age ranged from 17 to 22, and the average age was 19.07 ± 1.09 years. The bedtime procrastination of junior college nursing students by demographic characteristics is shown in Table 1. Ggrade and monthly household income are significantly related to bedtime procrastination.

**Table 1:** Demographic characteristics and single factor analysis of bedtime procrastination of junior college nursing students (*n* = 1827)

Factors	n(%)	Bedtime Procrastination Mean ± SD	t/F	***p-value***
Gender				
Male	447 (24.50%)	24.57 ± 6.88	−1.974*	0.056
Female	1380 (75.50%)	25.29 ± 6.87		
Age				
≤ 18	569 (31.10%)	24.74 ± 6.96	0.945**	0.418
19	671 (36.70%)	25.33 ± 6.91		
20	426 (23.30%)	25.32 ± 6.77		
≥ 21	161 (8.80%)	24.98 ± 6.79		
Grade				
First-Year	1286 (70.40%)	24.81 ± 6.78	5.285**	0.005
Second-Year	420 (23.00%)	26.07 ± 7.10		
Junior-Year	121 (6.60%)	24.98 ± 6.97		
Family location				
City	664 (36.30%)	25.39 ± 7.33	1.297*	0.195
Rural	1163 (63.70%)	24.96 ± 6.61		
Number of sibling				
Zero	1014 (55.50%)	25.29 ± 6.94	1.215*	0.224
At least one	813 (44.50%)	24.90 ± 6.81		
Monthly household income				
<3000RMB	940 (51.50%)	24.64 ± 6.51	4.933**	0.007
3000 ~ 6000RMB	704 (38.50%)	25.53 ± 7.09		
>6000RMB	183 (10.00%)	25.95 ± 7.72		

*t, **F

### Correlation between the degree of bedtime procrastination in junior college nursing students and influencing factors

The correlation matrix shows that the five personalities and future time perspective were significantly negatively correlated (*p* < 0.01) with bedtime procrastination. Self-regulatory fatigue and problematic mobile phone use were positively correlated with the degree of bedtime procrastination (*p* < 0.01) (Table 2).

**Table 2:** Correlation analysis of factors influencing bedtime procrastination of junior college nursing students (*n* = 1827)

Items	Mean ± SD	Bedtime Procrastination
Bedtime Procrastination	25.11 ± 6.88	1
Extroversion	8.79 ± 2.40	− 0.075**
Agreeableness	10.18 ± 2.12	−0.230**
Conscientiousness	9.56 ± 2.21	−0.323**
Neuroticism	9.05 ± 2.26	−0.334**
Openness	9.39 ± 2.09	−0.237**
Cognitive Subscale	16.76 ± 2.70	0.363**
Behavioral Subscale	12.79 ± 4.62	0.315**
Emotional Subscale	12.92 ± 3.67	0.388**
Self-regulatory Fatigue	42.47 ± 9.46	0.408**
Behavioral Commitment	11.01 ± 2.86	−0.300**
Far-reach Goal Orientation	7.91 ± 0.89	−0.208**
Future Efficacy	11.18 ± 3.02	−0.227**
Future Purpose Consciousness	13.34 ± 1.84	−0.264**
Future Image	11.39 ± 2.22	−0.244**
Future Time Perspective	54.82 ± 8.46	−0.326**
Thirst	6.18 ± 3.10	0.233**
Withdrawal	12.70 ± 6.21	0.280**
Reaction of Body and Mind	8.69 ± 4.24	0.335**
Problematic Mobile Phone Use	27.57 ± 12.58	0.309**

*Note* **p<0.01

### Multiple linear regression of bedtime procrastination of junior college nursing students

Multiple linear regression models (α_in_ = 0.05, α_out_ = 0.10) were fitted with bedtime procrastination scores as dependent variables and statistically significant general data in the correlation analysis as independent variables. The assignment of independent variables is shown in Table 3. The results showed that 7 factors were included in the multiple linear regression model, namely, monthly household income, extroversion, conscientiousness, neuroticism, self-regulatory fatigue, future time perspective, and problematic mobile phone use (Additional file 2), which together explained 21.7% of the total variance in junior college nursing students’ bedtime procrastination (Table 4).

**Table 3:** Table of independent variable assignment

Independent variable	Assignment (Dummy coded)
Grade	First-Year(Z1 = 0,Z2 = 0,Z3 = 0),Second-Year(Z1 = 0,Z2 = 1,Z3 = 0),Junior-Year (Z1 = 0,Z2 = 0,Z3 = 1)
Monthly household income	<3000RMB (Z1 = 0,Z2 = 0,Z3 = 0),3000 ~ 6000RMB(Z1 = 0,Z2 = 1,Z3 = 0),>6000RMB (Z1 = 0,Z2 = 0,Z3 = 1)
Extroversion	Bring in the original scores
Agreeableness	Bring in the original scores
Conscientiousness	Bring in the original scores
Neuroticism	Bring in the original scores
Openness	Bring in the original scores
Self-regulatory Fatigue	Bring in the original scores
Future Time Perspective	Bring in the original scores
Problematic Mobile Phone Use	Bring in the original scores

**Table 4:** Multiple linear regression analysis of the influencing factors of the bedtime procrastination of junior college nursing students (*n* = 1827)

Item	B	SE	β	t	***P***
Constant	25.747	2.033		12.667	<0.001
Monthly household income(<3000RMB = contrast)					
3000 ~ 6000RMB	0.740	0.305	0.052	2.429	0.015
>6000RMB	1.708	0.496	0.075	3.442	0.001
Extroversion	0.225	0.066	0.078	3.406	0.001
Conscientiousness	−0.284	0.096	− 0.091	−2.961	0.003
Neuroticism	−0.203	0.094	−0.067	−2.157	0.031
Self-regulatory Fatigue	0.135	0.024	0.186	5.555	<0.001
Future Time Perspective	−0.141	0.021	−0.173	−6.543	<0.001
Problematic Mobile Phone Use	0.078	0.014	0.143	5.667	<0.001

R = 0.471, R^2^ = 0.222, Adjusted R^2^ = 0.217, F = 43.181, *P*<0.001

## Discussion

### Bedtime procrastination in junior college nursing students

In recent years, sleep problems among nursing students have received increasing attention from scholars, but studies related to bedtime procrastination in this population are limited. In the present study, the total bedtime procrastination score of nursing students was 25.11 ± 6.88, indicating that bedtime procrastination among the included Chinese junior college nursing students was at an moderate level. Several previous studies [19, 33] have examined the level of bedtime procrastination among nursing students, and their findings are not consistent with ours. The reasons for this may be as follows: first, the nursing students evaluated were from different countries with economic and cultural differences; second, the measurement instruments used were different. For these reasons, the findings in our study differed from those of previous studies.

### Influencing factors of bedtime procrastination

We found that bedtime procrastination among nursing students with monthly household incomes of 3000–6000 RMB and > 6000 RMB was worse than among those with monthly household incomes < 3000 RMB, which is an interesting finding that has not been reported in the previous literature. In general, the higher the monthly household income, the higher the monthly cost of living for students and the more financially capable they are of engaging in recreational activities or activities of interest. Kroese et al. [34] demonstrated that most people delay sleep behavior due to immersive activities that encroach on sleep before bedtime. If a junior college nursing student has a passion for a particular hobby (e.g., art, music, or painting) and has the financial means to do so, they will likely become immersed in that activity and ignore the passage of time, which could lead to bedtime procrastination.

Personality is a stable trait in individuals, and the Big Five is one of the most recognized and widespread theories in the field related to personality. Previous studies [35] have found a significant relationship between personality and general procrastination. Our study showed that the more pronounced the extroverted personality among junior college nursing students, the more severe the degree of bedtime procrastination. Extroversion personality is characterized by enthusiasm, activity, and risk-taking, as well as a high degree of gregariousness. Nursing students with extroverted personalities are likely to be lively and get along with people and are more likely to spend much of their time with others, including at night, thus contributing to bedtime procrastination. The present study showed that high levels of conscientiousness reduced bedtime procrastination, which is consistent with previous research findings [21]. Conscientiousness can motivate individuals to be persistent, self-disciplined, and to seek achievement. Nursing students with high levels of self-discipline can complete their study tasks efficiently and on time, which means they do not have to delay sleep to complete unfinished work at night. In addition, previous research [36] has shown that conscientious, persistent people are more likely to pursue long-term goals, so they do not delay going to bed to avoid reducing their productivity during the day. Our study showed that neuroticism may also reduce bedtime procrastination in junior college nursing students. A neurotic personality is generally characterized by irritability, emotional instability, and poor control of negative emotional reactions. Previous studies have found that a neurotic personality is significantly associated with daytime fatigue, so we speculate that daytime fatigue in nursing students with neurotic personality are too tired to delay their bedtime and instead use good sleep to relieve fatigue.

Our study found that self-regulatory fatigue was a significant positive predictor of bedtime procrastination in junior college nursing students, which is consistent with previous research findings [23]. According to the strength model of self-control [37], an individual’s self-control is based on a limited mental resource, and all activities requiring self-control consume the same self-control resource. Self-regulatory fatigue occurs when the self-control resource is depleted and the individual is unable to exercise good self-control. When nursing students carry out self-control activities during the day, such as active studying, taking exams, or participating in social activities such as clubs, they do not have sufficient self-control resources before going to bed and are unwilling to control themselves in going to bed on time, leading to bedtime procrastination. It has been suggested that effective measures are needed to alleviate the level of self-regulatory fatigue among junior college nursing students and retain self-control resources to alleviate levels of bedtime procrastination.

Previous studies have found a significant relationship between future time perspective and general procrastination [38] and academic procrastination [39]. We also found that future time perspective can reduce bedtime procrastination in junior college nursing students. Future time perspective, which is expressed in the individual’s perception, emotional experience, and action (action tendency) regarding the future, can help nursing students to be confident in using future time and responding to future events and plan rationally in terms of academic and life aspects such as bedtime, thus avoiding bedtime procrastination [40]. What’s more, existing studies [41] have revealed a neural basis of future time perspective. Future time perspective is significantly and negatively correlated with the volume of gray matter in the parahippocampal gyrus and ventral medial prefrontal cortex. Both parahippocampal gyrus and ventral medial prefrontal cortex gray matter are significantly associated with procrastination.

We found that problematic mobile phone use significantly increased bedtime procrastination among junior college nursing students. Previous studies [42, 43] have generally confirmed this finding. In China, people are highly dependent on smartphones, relying on them for work, study, socializing, payments, shopping, communication, and even health records. Nearly everyone in the country has a smartphone. In addition to various essential functions, smartphones are also filled with various types of entertainment: games, videos, and so on. The excessive use of smartphones at bedtime by junior college nursing students leads to indulgence in entertainment activities and failure to go to bed on time, thereby causing bedtime procrastination. Nursing educators and nursing schools can guide nursing students in the appropriate use of smartphones to help alleviate bedtime delays.

### Practical implications

This was the first study to assess bedtime procrastination and influencing factors among Chinese junior college nursing students. Our findings can inform nursing schools, educators, and parents regarding bedtime procrastination among nursing students to help develop strategies to alleviate the level of bedtime procrastination. Such strategies should focus on household monthly income levels, internal traits such as personality, self-regulatory fatigue, future time perspective, and problematic mobile phone use.

### Strengths and limitations

This study has several strengths. First, this research is the first to investigate bedtime procrastination among Chinese junior college nursing students. The published literature on bedtime procrastination among college nursing students is scarce. Thus, our study makes an important contribution to the literature. Second, this study explored the factors influencing bedtime procrastination among junior college nursing students. Nursing schools and nursing educators can use these findings to help students address bedtime procrastination.

However, there are also some limitations to this study. First, due to the cross-sectional study design, we could not establish any causal relationships. Second, because this study was conducted at only one institution rather than a random selection from each province in China, there is the possibility of selection bias. Finally, we only included junior college nursing students, and omitted undergraduate and graduate students; therefore, the findings are not representative of all nursing students. Further research is needed to determine whether these findings can be generalized to other higher education settings.

## Conclusions

In this study, we explored the current status of bedtime procrastination among junior college nursing students and its influencing factors. Bedtime procrastination among college nursing students was at a moderate level, and the main influencing factors were monthly household income; extroversion, conscientiousness, and neuroticism; self-regulation fatigue; a future time perspective; and problematic mobile phone usage. Larger samples recruited from different schools and regions should be included in future research, and structural equation modeling is recommended for analyzing the relationship between bedtime procrastination and influencing factors. Nursing educators should pay greater attention to the problem of bedtime procrastination among nursing students. Future longitudinal and intervention studies are required to confirm whether interventions based on related factors contribute to alleviating the degree of bedtime procrastination to protect the physical and mental health of nursing students and enable them to successfully complete their studies.

## Supplementary Information

**Additional file 1. **Questionnaire of factors influencing bedtime procrastination in junior college nursing students (English translation).

**Additional file 2. **Scatter diagram of bedtime procrastination in junior college nursing students (English translation).

**Additional file 3.**

## Data Availability

All data generated or analysed during this study are included in this published article and its supplementary information files.

## Abbreviation

COVID-19: Corona virus disease 2019
