# Supplementary material for: Factors influencing bedtime procrastination in junior college nursing students: a cross-sectional study
Source: BMC Nurs. 2022 Apr 27;21:97. doi: 10.1186/s12912-022-00881-7 (PMC9042658; doi:10.1186/s12912-022-00881-7)
Supplement: Supplementary file 1 — Additional file 1. Questionnaire of factors influencing bedtime procrastination in junior college nursing students (English translation). [file 12912_2022_881_MOESM1_ESM.pdf]

Supplementary file 1

Questionnaire of factors influencing bedtime procrastination in junior college nursing students(English translation)

1. Your gender: Male/female
2. Your age: 17 18 19 20 21 22
3. Grade: Freshman, sophomore and junior
4. Family location: urban and rural areas
5. Number of sibling: Zero At least one
6. Monthly household income: < 3000 3000-6000 > 6000

ITEMS	Never	occasionally	Sometimes	Usually	Always
7.1 I went to bed later than planned	1	2	3	4	5
7.2 If I have to get up early the next day, I will go to bed early	1	2	3	4	5
7.3 If it is time to go to bed at night, I will go to bed immediately	1	2	3	4	5
7.4 Often, when it's time to go to bed, I'm still doing other things	1	2	3	4	5
7.5When I really want to go to sleep, I tend to be distracted by other things and do not go to sleep	1	2	3	4	5
7.6I didn't go to bed on time	1	2	3	4	5
7.7 I keep a regular bedtime	1	2	3	4	5
7.8I want to go to bed on time, but I can't	1	2	3	4	5
7.9 When it's time to go to bed, I can easily stop what I'm doing and go to sleep	1	2	3	4	5

ITEMS	Absolutely disagree	Strongly disagree	Basically disagree	Not sure	Basically agree	Strongly agree	Absolutely agree
8.1 Outgoing and energetic	1	2	3	4	5	6	7
8.2 Introverted and quiet	1	2	3	4	5	6	7
8.3 Being critical or quarrelsome	1	2	3	4	5	6	7
8.4 Pleasant and friendly	1	2	3	4	5	6	7
8.5 Reliable and self-disciplined	1	2	3	4	5	6	7
8.6 Poorly organized or careless	1	2	3	4	5	6	7
8.7Worried or easily upset	1	2	3	4	5	6	7
8.8 Calm and emotionally stable	1	2	3	4	5	6	7
8.9 Open to new things, often with new ideas	1	2	3	4	5	6	7
8.10 Conforming to conventions and not being innovative	1	2	3	4	5	6	7

ITEMS	Absolutely	Disagree	Not sure	Agree	Absolutely

	disagree				agree
9.1 I feel energetic	1	2	3	4	5
9.2 I can set goals easily	1	2	3	4	5
9.3It is difficult for me to carry out my exercise plan	1	2	3	4	5
9.4 Making decisions is not a problem for me	1	2	3	4	5
9.5It's easy for me to keep in touch with my friends	1	2	3	4	5
9.6 I have trouble remembering something	1	2	3	4	5
9.7 I have had the urge to destroy something	1	2	3	4	5
9.8 I'm always thinking unpleasant things	1	2	3	4	5
9.9 I try to avoid discussing or thinking about what bothers me	1	2	3	4	5
9.10I can't control my temper	1	2	3	4	5
9.11 I have had the urge to hit or hurt people	1	2	3	4	5
9.12 I get frustrated easily	1	2	3	4	5
9.13I handle pressure well	1	2	3	4	5
9.14 I cry easily	1	2	3	4	5
9.15It's easy for me to stick to a healthy diet	1	2	3	4	5
9.16 I'm always moody	1	2	3	4	5

ITEMS	completely noncompliant	A little compliant	compliant	fully compliant
10.1 I have goals to strive for every day	1	2	3	4
10.2 Once I have determined my goal, I will take specific measures to achieve it	1	2	3	4
10.3 Once I have decided what to do, I consider how to accomplish it	1	2	3	4
10.4 I accomplished the plan on time by progressing gradually	1	2	3	4
10.5I believe I have the ability to build my own beautiful tomorrow	1	2	3	4
10.6 I think my future is bright	1	2	3	4
10.7I am full of confidence in my future	1	2	3	4
10.8 I often remind myself not to forget the most important goal in the future	1	2	3	4
10.9 I often think about what I want to achieve in five years' time	1	2	3	4
10.10 I evaluate information about life development in terms of whether it contributes to long-term goals	1	2	3	4
10.11 I often imagine how I will change in the course of my life	1	2	3	4
10.12 I often ask myself what is my long-term goal in life	1	2	3	4
10.13 The course of my life is determined by forces beyond my control	1	2	3	4

10.14 I often feel that life has no purpose	1	2	3	4
10.15 I am quite concerned about negative comments on my future development	1	2	3	4
10.16I have a very vague idea of my future	1	2	3	4
10.17 I believe that my future is largely determined by fate	1	2	3	4
10.18Looking ahead, I have a lot of things to do	1	2	3	4
10.19 What do I know is my main task right now	1	2	3	4
10.20I know there are many tasks to be done in the future	1	2	3	4

ITEMS	Never	Occasionally	Sometimes	Usually	Always
11.1 I never feel like I spend enough time on my phone	1	2	3	4	5
11.2 I think I need to spend more time on the phone to be satisfied	1	2	3	4	5
11.3 I often have dreams about mobile phones	1	2	3	4	5
11.4 When I try to reduce or stop using my phone, I feel depressed, depressed, or irritable	1	2	3	4	5
11.5 Irritability occurs when you have to turn off your cell phone for class, dinner, etc	1	2	3	4	5
11.6 When my phone has not rung for a while, the thought of a missed call begins to cross my mind	1	2	3	4	5
11.7 When I hear my phone ring or vibrate, I automatically take out my phone to check	1	2	3	4	5
11.8 I feel anxious when I do not check my phone for messages or turn it off for a period of time	1	2	3	4	5
11.9 I would feel lost without my cell phone	1	2	3	4	5
11.10 I don't get enough sleep because I use my phone too much	1	2	3	4	5
11.11 I sometimes prefer to play with my phone rather than deal with other more pressing matters	1	2	3	4	5
11.12Due to the use of mobile phone, MY leisure time is reduced	1	2	3	4	5
11.13 Using mobile phones directly affects my study or work efficiency	1	2	3	4	5
