# Supplementary material for: Factors influencing bedtime procrastination in junior college nursing students: a cross-sectional study
Source: BMC Nurs. 2022 Apr 27;21:97. doi: 10.1186/s12912-022-00881-7 (PMC9042658; doi:10.1186/s12912-022-00881-7)

Supplementary file 2

Scatter diagram of bedtime procrastination in junior college nursing students(English translation)

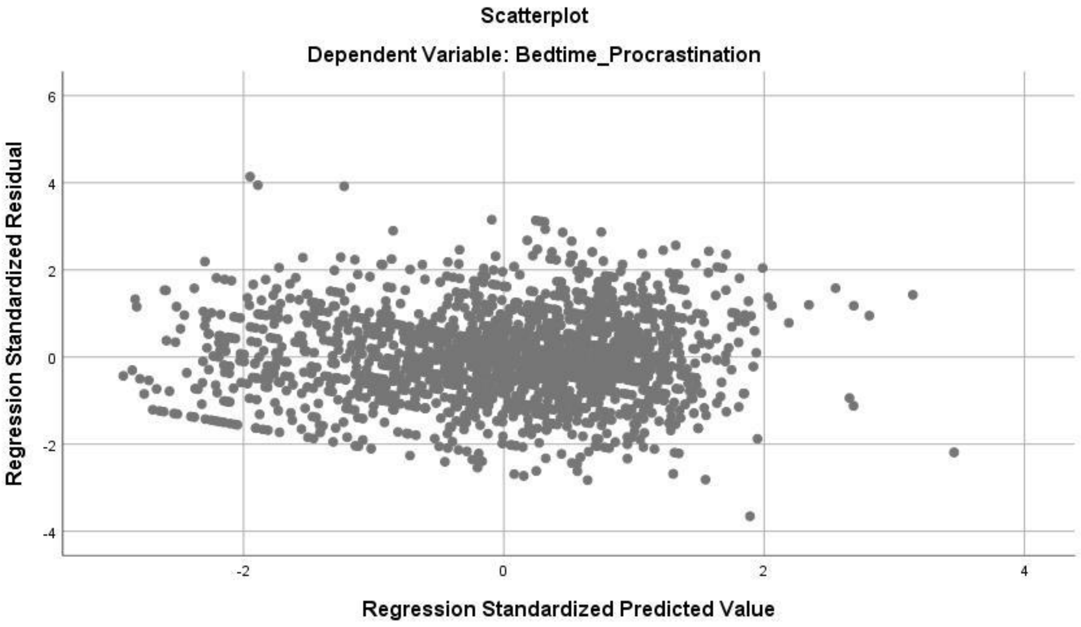

Supplement: Supplementary file 2 — Additional file 2. Scatter diagram of bedtime procrastination in junior college nursing students (English translation). [file 12912_2022_881_MOESM2_ESM.pdf]
